# Transition of allele-specific DNA hydroxymethylation at regulatory loci is associated with phenotypic variation in monozygotic twins discordant for psychiatric disorders

**DOI:** 10.1186/s12916-023-03177-y

**Published:** 2023-12-12

**Authors:** Junping Ye, Zhanwang Huang, Qiyang Li, Zhongwei Li, Yuting Lan, Zhongju Wang, Chaoying Ni, Xiaohui Wu, Tingyun Jiang, Yujing Li, Qiong Yang, Junghwa Lim, Cun-Yan Ren, Meijun Jiang, Shufen Li, Peng Jin, Jian-Huan Chen, Cunyou Zhao

**Affiliations:** 1 Key Laboratory of Mental Health of the Ministry of Education, Guangdong-Hong Kong-Macao Greater Bay Area Center for Brain Science and Brain-Inspired Intelligence, Guangdong Province Key Laboratory of Psychiatric Disorders, and Department of Medical Genetics, School of Basic Medical Sciences, Southern Medical University, Guangzhou, 510515 China; 2 Department of Rehabilitation, Zhujiang Hospital of Southern Medical University, Guangzhou, China; 3 Department of Psychiatry, the Affiliated Brain Hospital of Guangzhou Medical University (Guangzhou Huiai Hospital), Guangzhou, Guangdong China; 4 The Third People’s Hospital of Zhongshan, Zhongshan, Guangdong China; 5 Departments of Human Genetics, Emory University, Atlanta, GA USA; 6 Laboratory of Genomic and Precision Medicine, Wuxi School of Medicine, Jiangnan University, Wuxi, China; 7 Guangdong Provincial People’s Hospital (Guangdong Academy of Medical Science), Guangdong Mental Health Center, Southern Medical University, Guangzhou, China; 8 Experimental Education/Administration Center, School of Basic Medical Science, Southern Medical University, Guangzhou, China

**Keywords:** Schizophrenia, Bipolar disorder, 5hmC, Monozygotic twins, Allele-specific hydroxymethylation, *PLLP*

## Abstract

**Background:**

Major psychiatric disorders such as schizophrenia (SCZ) and bipolar disorder (BPD) are complex genetic mental illnesses. Their non-Mendelian features, such as those observed in monozygotic twins discordant for SCZ or BPD, are likely complicated by environmental modifiers of genetic effects. 5-Hydroxymethylcytosine (5hmC) is an important epigenetic mark in gene regulation, and whether it is linked to genetic variants that contribute to non-Mendelian features remains largely unexplored.

**Methods:**

We combined the 5hmC-selective chemical labeling method (5hmC-seq) and whole-genome sequencing (WGS) analysis of peripheral blood DNA obtained from monozygotic (MZ) twins discordant for SCZ or BPD to identify allelic imbalances in hydroxymethylome maps, and examined association of allele-specific hydroxymethylation (AShM) transition with disease susceptibility based on Bayes factors (BF) derived from the Bayesian generalized additive linear mixed model. We then performed multi-omics integrative analysis to determine the molecular pathogenic basis of those AShM sites. We finally employed luciferase reporter, CRISPR/Cas9 technology, electrophoretic mobility shift assay (EMSA), chromatin immunoprecipitation (ChIP), PCR, FM4-64 imaging analysis, and RNA sequencing to validate the function of interested AShM sites in the human neuroblastoma SK-N-SH cells and human embryonic kidney 293T (HEK293T) cells.

**Results:**

We identified thousands of genetic variants associated with AShM imbalances that exhibited phenotypic variation-associated AShM changes at regulatory loci. These AShM marks showed plausible associations with SCZ or BPD based on their effects on interactions among transcription factors (TFs), DNA methylation levels, or other epigenomic marks and thus contributed to dysregulated gene expression, which ultimately increased disease susceptibility. We then validated that competitive binding of POU3F2 on the alternative allele at the AShM site rs4558409 (G/T) in *PLLP*-enhanced *PLLP* expression, while the hydroxymethylated alternative allele, which alleviated the POU3F2 binding activity at the rs4558409 site, might be associated with the downregulated *PLLP* expression observed in BPD or SCZ. Moreover, disruption of rs4558409 promoted neural development and vesicle trafficking.

**Conclusion:**

Our study provides a powerful strategy for prioritizing regulatory risk variants and contributes to our understanding of the interplay between genetic and epigenetic factors in mediating SCZ or BPD susceptibility.

**Supplementary Information:**

The online version contains supplementary material available at 10.1186/s12916-023-03177-y.

## Background

Major psychiatric disorders, including schizophrenia (SCZ) and bipolar disorder (BPD), are complex genetic mental illnesses that affect more than 1% of people worldwide [1]. The complexity of these disorders arises from both their heterogenetic symptoms and multifactorial: genetic, developmental, and environmental nature. Epidemiological and genetic studies have indicated substantial overlap and high relative risks among relatives of both SCZ and BPD patients [1]. Numerous genetic variants in noncoding regions implicated by genome-wide association studies (GWAS) are enriched in regulatory domains [2], indicating that regulatory role for nonsequence-based genomic variation in mediating associations among genetic risk burden, environmental or epigenetic risk exposure, and phenotype. Recently, the identification of nonimprinted allele-specific DNA methylation (ASM) associated with genetic variation in *cis,* where the genetic variant was associated with DNA methylation of a neighboring cytosine base on the same chromosome, suggested that mapping this type of allelic asymmetry may prove useful as a post-GWAS strategy for identifying functional genetic variants [3–5]. Our recent study based on monozygotic (MZ) twins discordant for SCZ or BPD revealed thousands of single-nucleotide polymorphisms (SNPs) identified with ASM imbalances and phenotypic variation-associated switching at regulatory loci and that affected the interaction among one or more transcription factors (TFs), DNA methylation levels, and likely other epigenomic mark levels [3]. Mapping of ASM or allele-specific chromatin states has also been reported to facilitate genome-wide screening for disease-linked regulatory SNPs [4, 6, 7], which can be prioritized for functional studies. Since there are two alleles on homologous chromosomes in an individual in the same cellular and nuclear environment, there might be heterogeneity between allelic epigenomic patterns among individuals, providing important implications for interindividual differences in disease susceptibility.

Recently, 5-hydroxymethylcytosine (5hmC), which is catalyzed from 5mC by ten eleven translocation (TET) proteins, was discovered to be a relatively abundant form of cytosine modification in embryonic stem cells (ESCs) and Purkinje neurons [8, 9]. This discovery raised the possibility that 5hmC mark may function as an intermediate during DNA demethylation; in addition, it may be a regulatory epigenetic mark that alters chromatin structure or contributes to the recruitment or exclusion of other DNA-binding proteins that affect transcription, and therefore, the dysregulation of this mark may cause certain diseases, such as SCZ or BPD [10, 11]. Previously, sequence-dependent allelic imbalances in the epigenome, including imbalanced DNA methylation and histone marks or open chromatin at regulatory loci, have been reported to cause disease-associated switching, providing a powerful framework for identifying disease-associated variants and genes [3, 4, 7]. Although several groups have investigated the genomic distribution of DNA hydroxymethylation marks [12, 13], the role and functional importance of this modification and its links to disease-associated genetic variants in SCZ or BPD are still unclear.

We recently studied MZ twin epigenetic profiles and observed epigenetic variations, such as differences in DNA methylation [3], lncRNA [14], and miRNA [15] levels, in phenotypes among concordant or discordant MZ twins. Examination of the epigenetic profiles of MZ twins, particularly disease-discordant MZ twins, is a powerful strategy to gain understanding of how genetic, environmental, and stochastic factors impact epigenetic modifications and how epigenetic variations affect the acquisition of complex traits [3, 16]. MZ twins were exclusively matched for genotype, age, sex, paternal effects, population cohort effects, and exposure to several shared environmental factors. In summary, the same sources of cells from MZ cotwins were expected to exhibit identical genetic signatures, and alterations in allele-specific epigenetic modification in MZ cotwin pairs would indicate that certain epigenetic modifications may be particularly vulnerable to environmental influences or specific TFs, especially during embryonic development, leading to individual differences in phenotype and disease susceptibility [3, 14]. The effects of genetic variation on the stochasticity or metastability of the DNA hydroxymethylome on the underlying heterogeneity of human disease have been unexplored.

In this study, we combined the 5hmC-selective chemical labeling method (5hmC sequencing [5hmC-seq]) [17] and whole-genome sequencing (WGS) of peripheral blood DNA obtained from MZ twins discordant for SCZ or BPD to identify allelic imbalances in hydroxymethylome maps and found mechanistic effects of allele-specific hydroxymethylation (AShM) transitions (gain or loss) at regulatory loci on the phenotypic variation of psychiatric disorders in discordant MZ twins (PDC twins). Determining the molecular pathogenic basis of these differences will contribute to our understanding of the interaction between genetic and epigenetic factors in mediating individual differences in disease susceptibility and provide a powerful strategy for prioritizing SCZ/BPD-associated genes or variants.

## Methods

### Participants

A total of 14 pairs of MZ twins (28 individuals), including six PDC twin pairs (three SCZ-discordant (SDC) twins and three BPD-discordant (BDC) twins), four psychiatric disorder-concordant (PCC) twins (one SCZ-concordant (SCC) twin and three BPD-concordant (BCC) twins), and four healthy concordant (HCC) twins, was recruited in this study (Additional file 1: Table S1). The zygosity was determined by the Qiagen Investigator Argus X-12 QS Kit (USA). All patients fulfilled the diagnostic criteria of SCZ or BPD according to the Diagnostic and Statistical Manual of Mental Disorder, 4th Edition (American Psychiatric Association). All participants in this study provided informed consent prior to the study following presentation of the nature of the procedures. Approval for the study was obtained from the local medical ethics committee and conducted in accordance with the Declaration of Helsinki.

### WGS genotyping

To obtain genome-wide SNP genotype information, we performed WGS analysis of DNA from unaffected individuals of 14 MZ twin pairs with a depth of ~ 700 million 150-bp paired-end reads per sample on the Illumina Nova-seq by Novogene Solution (Tianjin, China). All sequencing data passed initial quality checks for base composition (no exclusions) using FASTQC and were mapped to hg19 using Bowtie2. We used Picard for removing duplicates and BaseRecalibrator and ApplyBQSR in GATK4 and public mutation datasets (1000G_phase1, Mills_and 1000G_gold_standard.indel, dbsnp_146) for validation of BAM files to obtain base quality scores. We used HaplotypeCaller in GATK4 for genotype determination and AlternativeRecalibrator and ApplyVQSR for cross-validation to obtain the final genotype of each SNP.

### Genome-wide analysis of AShM

We performed genome-wide DNA hydroxymethylation analysis using 5hmC-selective chemical labeling method (5hmC sequencing [5hmC-seq]) as previously described [12, 17]. Enriched DNA from 5hmC capture were subjected to library construction and to sequencing to a depth of ~ 26 million 51-bp single-end reads per sample on an illumine Hiseq by Annoroad Gene Technology (Beijing, China). All sequencing data passed initial quality checks for base composition (no exclusions) using FASTQC and was mapped to hg19 using Bowtie2 [18]. After removing duplicates using Picard, we quantified hydroxymethylation levels using MEDIPS [19] to produce the short read counts and the mean relative hydroxymethylation score (rhms) in each 500-bp bin (overlap of 250 bp) across the genome. The VarScan [20] package was used to detect the coverage, quality, and allele frequency of alternative allele from unique mapped data of 5hmC-seq followed by WGS genotyping. A genomic locus would be called out as a candidate polymorphic site if there was at least one high-quality read supporting the alternative allele. To reduce false positives, we only focused on candidate alternatives that had been called in WGS genotyping and showed concordant genotype in 28 MZ twins. We applied the Bayesian generalized additive linear mixed model approach (implemented using the INLA package) [21, 22] to quantify AShM based on 5hmC-seq read counts as described in our previous study [3]. For the individual AShM analyses, data were subset by AShM, and a binomial mixed model was fit with counts of reference and alternative reads as the outcome variable and random effects of disease status (*β*_*s*_) and twin subject (*γ*_*i*_): logit(*p*_*is*_)= *β*_0_ + *β*_*s*_ + *γ*_*i*_, where *p*_*is*_ is the proportion of reads supporting the alternative allele in twin *i* and disease status *s*. The posterior probability distribution of the intercept term (*β*_0_) was used to quantify the degree of AShM. Credible intervals were estimated by taking empirical quantiles of these distributions, and two-tailed posterior predictive *p* values were calculated based on the areas in the tail more extreme than the null alternative allele ratio of 0.5. Based on these *p* values, we used the Benjamini–Hochberg procedure to control the FDR at 10%. The SNP sites with FDR less than 0.1 were considered as the allele-specific hydroxymethylation bias sites (AShM sites). To validate the individual AShM sites that showed disease-associated AShM switching, we tested for each AShM whether a model including disease status as a fixed effect (*M*_1_: logit(*p*_*is*_)= *β*_0_ + *β*_*s*_ + *γ*_*i*_) fit the data significantly better than a reduced model without this term (*M*_0_: logit(*p*_*is*_)=*β*_0_ + *γ*_*i*_) among six PDC twins, where *p*_*is*_ is the proportion of reads supporting the alternative allele in twin *i* and disease status *s* and *γ*_*i*_ is the random effect of twin. We then compared the likelihoods of these two models to generate a Bayes factor (*BF*):

$${BF}_{\mathrm{1,0}}=\frac{p\left(\left.data\right|\mathrm{M}1\right)}{p\left(\left.data\right|\mathrm{M}0\right)}$$

We considered AShM sites with *BF* >1 as discordant AShM sites between affected and unaffected individuals, AShM sites with *BF* >10 were considered as discordant AShM sites with strong evidence of disease-associated AShM transition.

### Functional enrichment analysis of psyAShM sites

We used ANNOVAR software to annotate those alternatives called by Varscan and performed filter-based annotation and gene-based annotation to identify whether an alternative is reported in dbSNP and genomic context for each alternative. Gene Ontology term and pathway enrichment analysis was performed using the functional enrichment tool at https://toppgene.cchmc.org/enrichment.jsp [23].

To test enrichment of AShM sites among specific chromatin states, we obtained ten human brain tissues’ chromatin state from the chromHMM dataset using the 15-state model [24, 25]. The 15-state model consists of eight active states associated with gene expression (active TSS, TssA; flanking active TSS, TssAFlnk; transcription at 5ʹ and 3ʹends of genes, TxFlnk; strong transcription, Tx; weak transcription, TxWk; genic enhancers, EnhG; enhancers, Enh; Zinc finger genes/repeats, ZNF/Rpts) and seven repressed states (heterochromatin, Het; bivalent/poised TSS, TssBiv; flanking bivalent TSS/enhancer, BivFlnk; bivalent enhancer, EnhBiv; repressed Polycomb region, ReprPC; repressed PolyComb region ReprPCWk; quiescent, Quies). We calculated the number of SNPs located within each of 15 chromosome states for 807 psyAShM sites, and then enrichment was performed by testing whether the proportion of 807 psyAShM sites (Fisher’s exact test) within chromosome states were significantly higher compared with 117,012 AShM sites, and meta-analysis was performed to combine chromatin state enrichment results from different brain tissue enrichment results. Error bar plots were generated for visualization of meta results.

We then employed DeepSEA [26] to predict AShM sites occurrence within regulatory enhancer or promoter regions. An “e-value” is calculated by DeepsSEA based on the empirical distribution of that feature’s effect (abs(palt−pref)) among gnomAD alternatives to evaluate the expected proportion of SNPs with a larger predicted effect. We set an e-value of 0.01 as a significant effect, which indicates that only 1% of gnomAD alternatives have a larger predicted effect as a threshold to obtain a significant effect of SNPs on chromatin states. *Z*-score is a scaled score where the feature diff score (palt−pref) is divided by the root mean square of the feature diff score across gnomAD alternatives. Note that this is “sign-preserving”, i.e., a negative *z*-score indicates that a mutation decreases the probability of a regulatory feature. We focused on the *z*-scores of H3K27me3, H3K9me3, H3K4me3, H3K4me1, H3K27ac, and H3K36me3 histone markers in the human hippocampus, female fetal brain, and male fetal brain from DeepSEA [26], filtered alternatives outside the enhancer or promoters, and calculated the number of SNPs with the same or opposite direction as in 807 psyAShM sites. In detail, if SNPs whose alternative alleles have both positive/negative effects on enriched histone modifications of the above six histone markers while alternative allele frequency more/less than 0.5, the SNPs would be defined as having the same effect on histone modifications. Otherwise, the SNPs would be defined as having the opposite effect on histone modifications. Binom test is further used to test the consistency of effect direction for alternative allele on histone modification and DNA hydroxymethylation based on the binomial distribution.

We employed motifbreakR [27] to determine whether 807 psyAShM sites significantly overlapped a sequence match against a motif library of 431 human TF previously generated via the high-throughput systematic evolution of ligands by exponential enrichment method [28]. ScoreRef and scoreAlt were scores determined by the scoring method, when the sequence contains the reference/alternative SNP allele. The scores are scaled as a fraction of scoring range 0–1 of the motif matrix. We used feature’s effect difference (abs (scoreRef − scoreAlt)) among alleles to evaluate the effect of SNP on TF, then we analyzed the correlations between feature’s effect difference and hydroxymethylation levels of alternative allele with Pearson’s correlation (TF was considered significant correlation at *p*<0.05).

Four kinds of dataset, including SCZ+BPD, Alzheimer disease (AD), BPD, major depressive disorder (MDD), from transcriptome-wide association study (TWAS)-hub at http://twas-hub.org/ were used for enrichment analysis. The genes in these five gene sets were reported as potentially high risk genes related to SCZ and BPD, including activity-regulated cytoskeleton-associated protein (ARC), the N-methyl-d-aspartate receptor complex (NMDAR), fragile X mental retardation protein (FMRP) targets, postsynaptic density (PSD) proteins, and gamma-aminobutyric acid (GABA) [29–32]. Differential expressed genes were obtained from PsychENCODE consortium [33], CommonMind Consortium (CMC) [34], and lymphoblastoid cell lines (LCLs) [35]. We downloaded GTEx (Genotype-Tissue Expression) v8 eQTL dataset including 10 brain tissues, LIBD (Lieber institute for brain development) eQTL dataset including hippocampus and dorsolateral prefrontal cortex (DLPFC), and human brain mQTL dataset. GWAS datasets include PGC3 SCZ [36], PGC3 BPD [37], attention deficit hyperactivity disorder (ADHD) [38], and autism spectrum disorder (ASD) [39], and MDD [40]. We calculated the overlapping gene numbers with psyAShM genes and performed enrichment analysis with Fisher’s exact test using 150,000 SNP associated genes selecting by random sampling as background.

### Cell culture and luciferase reporter assay

The human SK-N-SH neuroblastoma cells, the most widely used system to study neurodevelopment [3, 41–45], have the ability to differentiate into a neuronal phenotype characterized by extensive neurite outgrowth, and were used to investigate the function of psyAShM sites in early neurodevelopmental processes and endocytic membrane trafficking. The cells were cultured in high-glucose Dulbecco’s modified Eagle’s medium (DMEM, Gibco) supplemented with 10% fetal bovine serum (FBS; FSP500, ExCell Bio) and maintained at 37°C with 5% CO_2_. For differentiation, SK-N-SH cells was induced with normal DMEM supplemented with 1.25% FBS and 10 μM all-trans retinoic acid (ATRA, Sigma) for 3 days after the cells had been seeded into a T175 flask in normal DMEM with 10% FBS, and the differentiation medium was changed every 24 h. The cells were photographed with an inverted fluorescent microscope (Nikon Ti-U, ×20). The neurite-like length was measured manually in a blinded manner with ImageJ software (https://imagej.nih.gov/ij/). We used the tool of “straight” to depict the neurite on SK-N-SH from the cell body to the end of neurite and the software would calculate the length through the bottom of analysis-measure. To examine the regulatory role of psyAShMs, we cloned DNA fragments spanning ~1000 bp upstream to ~600 bp downstream of psyAShM site identified in the enhancer region of a pGL4.23 luciferase reporter containing the minimal TATA-box promoter (minP). Mutations at the psyAShM site were achieved by using the site-directed mutagenesis method by replacing a major allele with a minor allele at the given SNP sites (Additional file 1: Table S2). These reporter constructs were transiently cotransfected into SK-N-SH or HEK293T cells together with the pRL-TK plasmid as an internal control for transfection efficiency using Lipofectamine 2000 reagents (11668019, Thermo Fisher). Cells were harvested 48 h after transfection, and the dual luciferase activity (Promega, E1960) was measured with the Victor X multilabel readers (PerkinElmer, San Jose, CA, USA).

### Electrophoretic mobility shift assay (EMSA), chromatin immunoprecipitation (ChIP), and PCR

Nuclear protein extraction from HEK293T cells was performed with EMSA to evaluate the affinity of allelic variations or hydroxymethylation alteration of rs4558409 (G/T) loci by using the Chemiluminescent Nucleic Acid Detection Module Kit (Thermo Fisher, USA). The biotin-labeled DNA complexes were visualized and quantified by using a chemiluminescence imaging system (Tanon 5200). ChIP assays were performed with cell extracts from HEK293T using anti-POU3F2 antibodies (1:50, Cell Signaling Technology, #12137). Cell lysates extracted from HEK293T cells were used with 2–4 μg antibodies for each assay and incubated overnight at 4°C. Protein G/Protein A agarose beads (Invitrogen™) were added for 3 h and quantified by qPCR (Additional file 1: Table S2).

### Lentivirus plasmid construction, packaging, and transduction

To examine the role of SNP rs4558409 in *PLLP*, we designed two sgRNA to disrupt this site from the genome based on CRISPR/Cas9 technology using online tools from the Zhang laboratory (https://zlab.bio/guide-design-resources). After synthesis from Invitrogen, the sgRNA oligonucleotides were cloned into a transfer lentiviral vector (lentiCRISPRv2, Addgene #52961) via a BsmBI site (NEB, SN: R0739). To package lentivirus, we cotransfected HEK293T cell with three plasmids (pSPAX2, Addgene#12259; pMD2G, Plasmid #12259 and lentiCRISPRv2 which carried sgRNA for *PLLP*) by polyethylenimine (YEASEN, SN: 40816ES02, 1 mg/ml). After 60 h transfection, the cell culture medium was collected and centrifuged at 10,000 rpm for 10 min, and the supernatant were further filtered with a 0.22-μm filter through the ultracentrifugal tubes (Crystalgen, SN: 23-2590) at 20,000 rpm for 2 h (Beckman Avanti J-E, rotor JA-20). The sediment was resuspended by 20 μl PBS and stored in −80℃. The SK-H-SN cells in a 12-well plate were infected with lentivirus. The cells were incubated for an additional 48–72 h prior to identification of GFP+ cells under an inverted fluorescence microscope, followed by screening for stable cell lines with 1 μg/ml puromycin (biosharp, SN: BS111-25mg). The effects of rs4558409 disruption on *PLLP* expression were confirmed by qRT-PCR and Western blotting with rabbit anti-PLLP (1:500, LifeSpan BioSciences LS-C398159) and mouse anti-GAPDH (1:50,000, Proteintech, 60004-1-Ig) as a loading control.

### FM4-64 imaging of SK-N-SH cells

We performed the experiments of endocytic membrane trafficking in SK-N-SH cells by FM4-64 unloading as described previously [46]. Culture medium of SK-N-SH cells were replaced with saline solution containing 170 mM NaCl, 3.5 mM KCl, 0.4 mM KH_2_PO_4_, 5 mM NaHCO_3_, 1.2 mM Na_2_SO_4_, 1.2 mM MgCl_2_, 1.3 mM CaCl_2_, 5 mM glucose, and 20 mM N-tris(hydroxymethyl)-methyl-2-aminoethane-sulfonic acid (pH 7.4) and incubated for 10 min. After that, the cells were loaded with 10 µM FM4-64 (Invitrogen) for 2 min in saline solution supplemented with 75 mM KCl. The cells were rinsed with saline solution only and then incubated with 10 µM FM4-64 in saline solution for 10 min. The cells were perfused three times with saline solution for a total of 5 min and then left in saline solution for an additional 10 min. FM4-64 imaging was performed on an LSM 880 with an Airyscan confocal microscope (Zeiss) with a C-Apochromat ×40/1.2 W Korr FCS M27 objective with images taken every 1.26 s at 25°C. A1-min baseline was recorded, followed by stimulation with 75 mM KCl in saline solution for 8 min. Cells were excited at 488 nm, and the emission was measured at 562 nm. The FM4-64 signal was determined by F = (F_1_/(F_0_ − B_0_). The signal was normalized to the mean fluorescence intensity measured at baseline.

### RNA-Seq analysis

RNA extracted from rs4558409-KO or wild-type SK-N-SH cell lines was employed for RNA-seq analysis on the Illumina Nova-PE150 by Novogene Solution (Tianjin, China) with a depth of ~30 million 150-bp paired-end reads per sample. The raw reads were subjected to quality control with FASTQC and then clean reads was aligned to the hg19 reference genome using HISAT2 with default parameters and converted the sam file to the bam file (binary format) using samtools. Read counts were derived from the GTF file generated by Stringtie with the parameters -A, -e and using the Python scripts provided by Stringtie. Differentially expressed genes (DEGs) were generated by DESeq2 in R. Gene Ontology (GO) analysis of significant genes were enriched using an online database (ToppGene) and visualized using the ggplot2 package in R.

## Results

### AShM sites showed SCZ/BPD-associated transitions in MZ twin pairs

**Fig 1:**
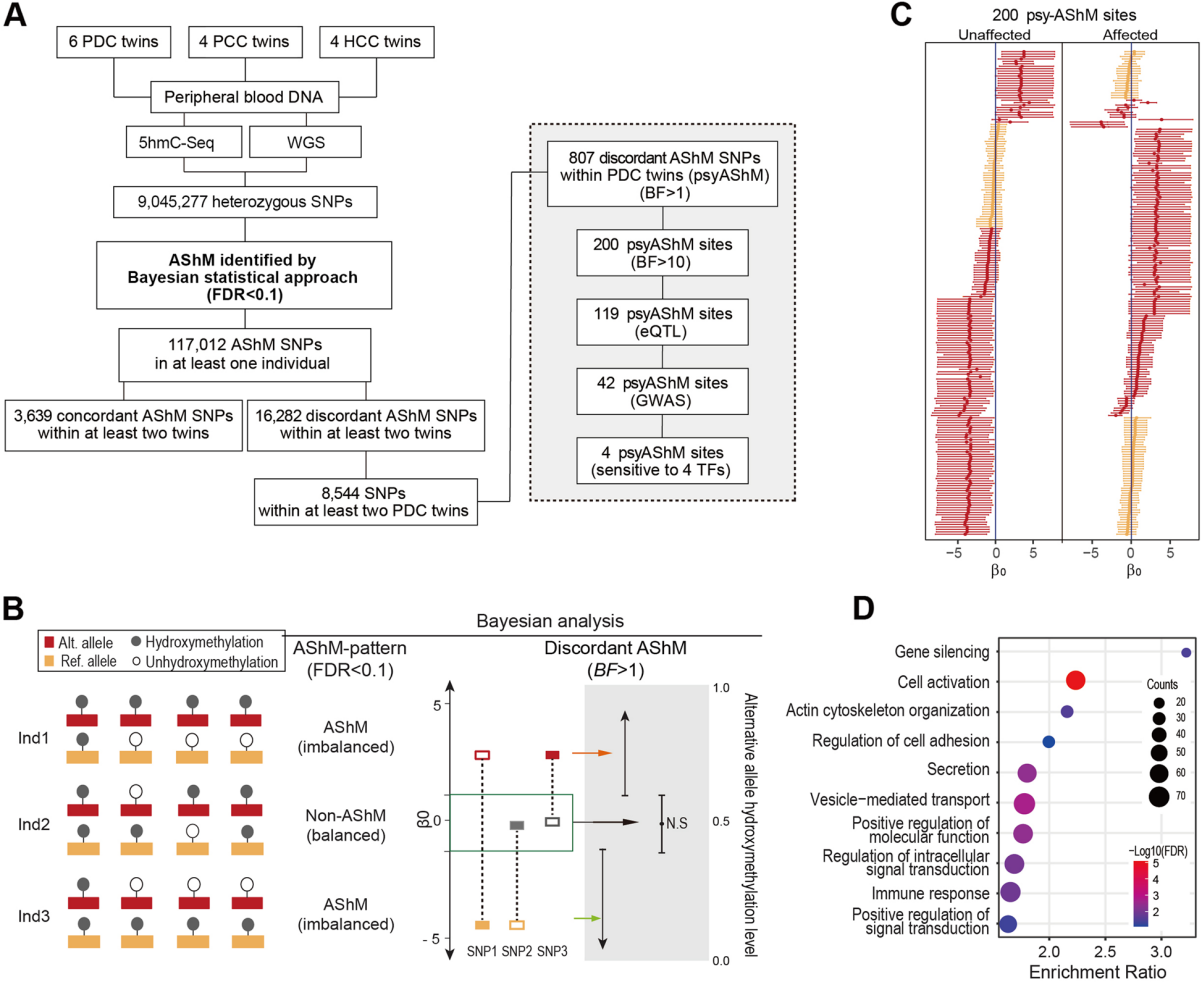
Schematic describing the method of detecting AShM in MZ twin pairs. **A** A schematic of the pipeline for the analysis of AShM. **B** Calling allelic imbalances. Allelic DNA hydroxymethylation patterns are shown in the left panel. Discordant AShM sites in twin pairs were determined by Bayesian analysis. **C** Estimates of 200 discordant AShM sites in PDC twin pairs. Each point represents the β0 value of the psyAShM sites showing the greatest evidence of AShM in unaffected or affected individuals (highlighted in red, *p*<1e−10) and the discordant AShM pattern within PDC twin pairs (BF>10). Error bars indicate 95% credible intervals. **D** Functional enrichment of GO-Biological process (GO-BP) annotations of 807 psyAShM sites

To identify individual SNP related to psychiatric disorder-associated AShM transitions, we performed 5hmC-seq (at a depth of ~ 26 million 51-bp single-end reads per sample) and WGS (at a depth of ~ 700 million 150-bp pair-end reads per sample; Fig. 1) with peripheral blood DNA obtained from 14 MZ twin pairs (28 individuals. Additional file 1: Table S1). We then performed a genome-wide DNA hydroxymethylation pattern evaluation with samples from the MZ twins, and observed that within-twin patterns of DNA hydroxymethylation levels were highly correlated among all MZ twin pairs (average r across all 500-bp windows within twins = 0.87; Additional file 2: Fig. S1), indicating high consistency in genome-wide DNA hydroxymethylation. We further evaluated the evidence for the sensitivity of the hydroxymethylome to genetic variation (viz. AShM) in 14 MZ twin pairs and discovered 9,045,277 informative SNPs with heterozygous genotypes in at least one twin pair, as determined by WGS genotyping. We used these data for further analysis of AShM on the alternative allele of each SNP (Fig. 1A and Additional file 1: Table S3). For each of these informative SNPs, we calculated a proportion of reads supporting evidence for an alternative allele in heterozygous individuals based on 5hmC-seq data via VarScan and then applied a Bayesian approach to assess DNA hydroxymethylation patterns exhibiting asymmetry between two alleles in each heterozygous individual (Fig. 1B). We discovered that 117,012 of 9,045,277 informative SNPs exhibited AShM patterns with a false discovery rate (FDR) criterion of 10% in at least one of 28 individuals; these SNPs included 53,425 AShM SNPs in one of the two individuals in at least one PDC twin pair, 50,967 SNPs in at least one PCC twin pair, and 45,068 SNPs in at least one HCC twin pair (Additional file 1: Table S3). Of the 117,012 identified AShM SNPs, 3639 SNPs exhibited concordant AShM patterns, and 16,282 SNPs exhibited discordant AShM patterns in two individuals in at least two twin pairs (Fig. 1A and Additional file 1: Table S3). Of the 117,012 identified AShM SNPs, 13,649 SNPs showed an ASM pattern (OR=5.84,*P* < 2.2e−16) as indicated in our recent report using data obtained from the same MZ twin cohort [3], and 534 AShM SNPs were located at 53 previously reported imprinted loci (Additional file 1: Table S4; https://www.geneimprint.org/). Moreover, eight AShM SNPs had exhibited allele-specific open chromatin (ASoC) in a previous assay for transposase-accessible chromatin using sequencing (ATAC-seq) [7]. These results support the idea that an allelic imbalance in the epigenome is a common phenomenon in the human genome and that the sensitivity to genetic variation or environmental influences that this epigenome imbalance confers may play roles in human diseases.

To examine whether AShM was associated with phenotype-associated transitions in the two individuals in a PDC twin pair, we applied Bayes factors (*BF*) derived from the Bayesian generalized additive linear mixed model [21, 22] to 8544 AShM SNPs to identify group-level AShM transition patterns among PDC twin pairs (Fig. 1B). We identified 807 SNPs (*BF*>1) and 200 SNPs (*BF*>10, Fig. 1C) that underwent a significant AShM transition from unaffected to affected individuals among the PDC twin pairs (viz. psychiatric disorder-associated AShM sites, psyAShM sites; Additional file 1: Table S5). Of the 807 identified psyAShM sites, 374 showed AShM on/off switching (199 with a gain of AShM and 175 with a loss of AShM), 177 SNPs showed AShM flipping from unaffected individuals to the affected individual among the PDC twins, and 256 SNPs showed similar AShM patterns between unaffected and affected individuals among the PDC twins. To describe the common features of the 807 psyAShM sites annotated in 484 unique genes, we performed Gene Ontology-Biological process (GO-BP) enrichment analysis with the 484 genes and identified a total of 41 GO-BP terms that passed a BH-adjusted *P* value<0.05 threshold of significance (Additional file 1: Table S6). The gene sets with the most enriched terms were involved in gene silencing, cell activation, actin cytoskeleton, regulation of cell adhesion, secretion, vesicle-mediated transport, positive regulation of molecular function, regulation of intracellular signal transduction, immune response, and positive regulation of signal transduction (Fig. 1D), part of which have been reported to be associated with SCZ/BPD [14, 46], providing functional implications for the psyASM sites in the development of these disorders.

### The psyAShM transition displays SCZ/BPD-associated features

**Fig 2:**
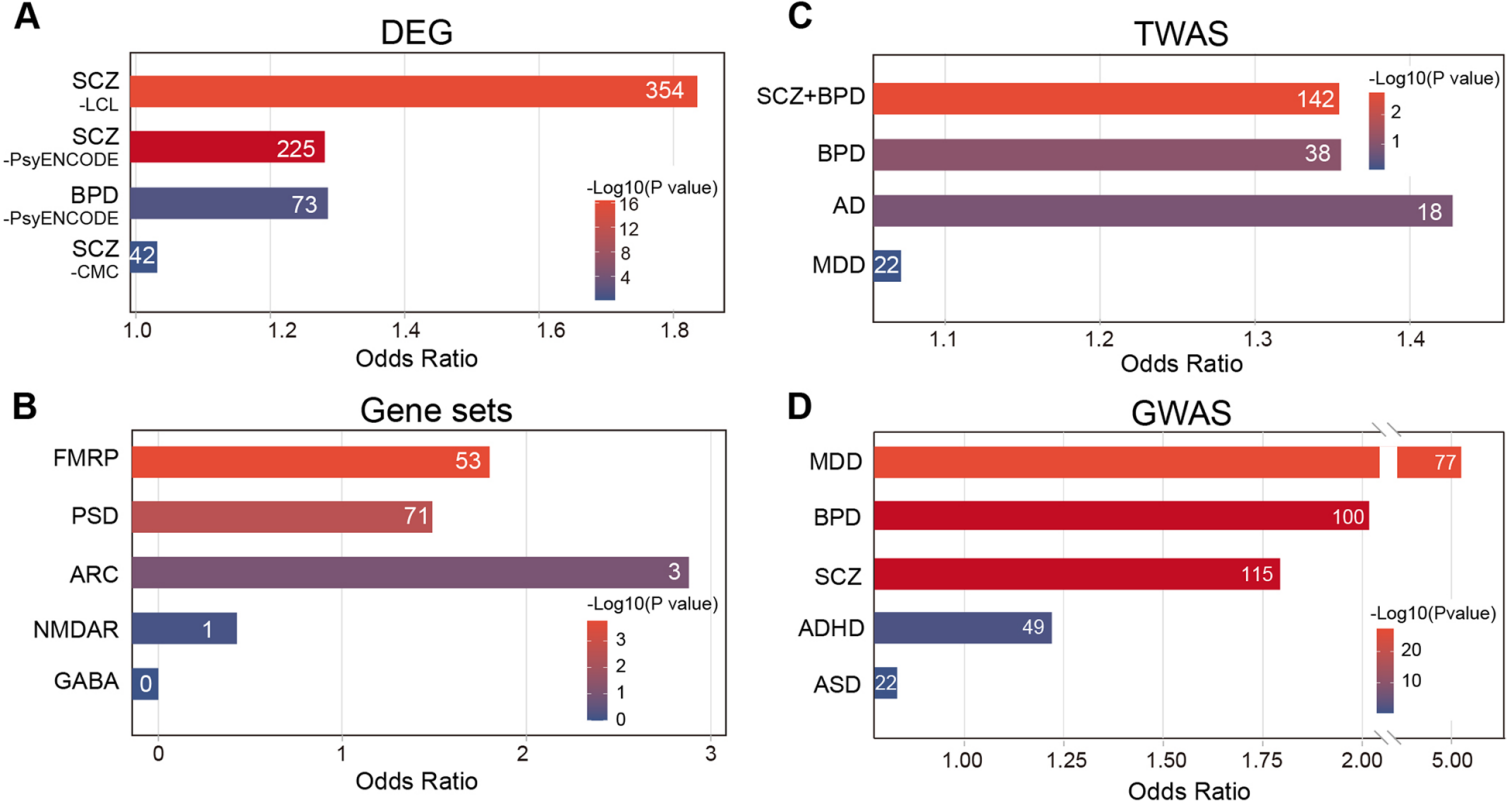
Disease-associated features of psyAShM genes.** A–D** Enrichment of 807 psyAShM sites in SCZ- or BPD-associated DEGs from the LCL, PsychENCODE, or CMC RNA-seq datasets (**A**), psychiatric disease-associated gene sets (**B**), TWAS (**C**), and GWAS (**D**) of SCZ, BPD, AD, MDD, ADHD, and ASD. The number of genes in each comparison is indicated

We then determined the genomic features of 807 psyAShM sites that are characteristic of SCZ or BPD risk genes. First, we observed that 807 psyAShM sites exhibited significant enrichment in lymphoblastoid cell lines (LCLs)-derived SCZ-associated DEGs (354 overlapping genes, odds ratio (OR) =1.84 and *P*=4.3e−17; Fig. 2A) [35], significant enrichment in brain-derived SCZ-associated DEGs (225 overlapping genes, OR=1.3 and *P*=0.0021), and marginal enrichment in brain-derived BPD-associated DEGs (73 overlapping genes, OR=1.3 and *P*=0.05) based on the PsychENCODE brain RNA-seq dataset [47]. Those brain-derived DEGs containing psyAShM sites, such as *PDE4B*, *RGS12*, *STXBP2*, and *SERPINE2*, are associated with cellular components of neuron projection, dendrite, synapse, postsynaptic density, and secretory vesicle, indicating the involvement of psyAShM transitions in the dysregulation of neuron-related gene expression in SCZ or BPD. We further assessed the enrichment of these pysAShM loci in two gene sets showing significantly enhanced ORs, including FMRP targets (*P*=1.7e−4, OR = 1.8) and PSD proteins (*P* =2.7e−3 and OR = 1.5; Fig. 2B), which have been previously implicated in SCZ or BPD [30, 31]. We also observed 807 psyAShM loci enriched significantly in a TWAS of SCZ+BPD patients (*P* =1.8e−3, OR = 1.4; Fig. 2C) [48]. These psyAShM sites were ultimately observed to be significantly enriched in subthreshold GWAS SNPs (*P*<0.05, Fig. 2D) of MDD (*P* =4.9e−28, OR=5.1), SCZ (*P* =6.1e−8, OR=1.8), and BPD (*P* =2.5e−8, OR=2.0) PGC GWAS loci [36–40]. Altogether, these results suggest that psyAShM loci are associated with psychiatric disorders and emphasize the value of using AShM data for prioritizing risk genes or variants.

### psyAShM transitions exerts epiallele-specific effects on chromatin states and TF binding affinities

**Fig 3:**
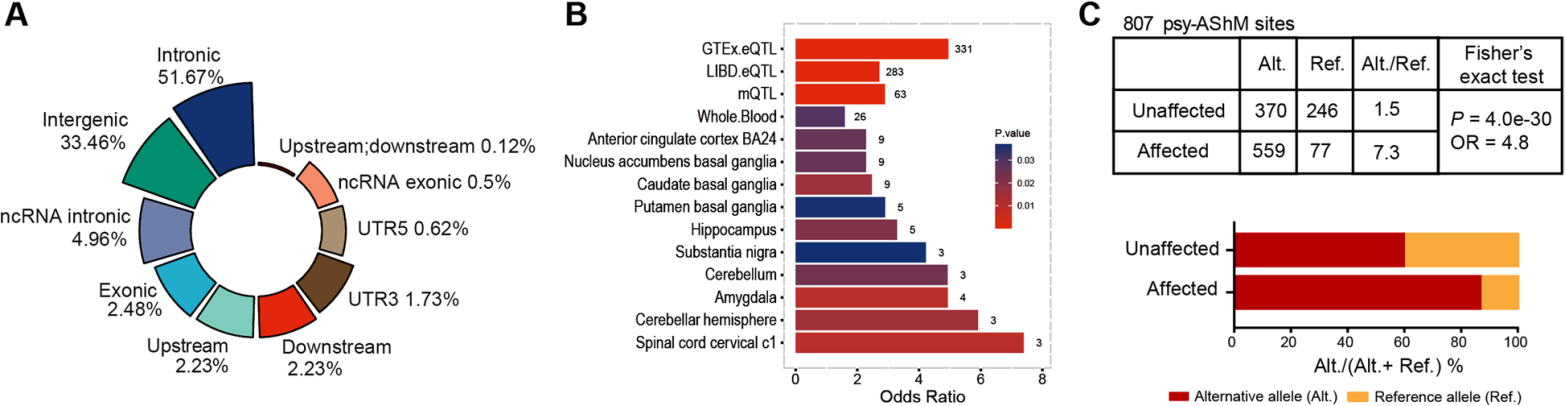
Characteristics of psyAShM sites. **A** The genomic context of 807 psyAShM sites. **B** Enrichment results of 807 psyAShM sites in eQTLs and brain mQTLs, and in genes expressed specifically in the brain or blood tissues. The number of genes in each comparison is indicated. **C** PsyAShM site counts showing preferential hydroxymethylation of alternative alleles in affected and unaffected individuals. The ratio is expressed as alternative/reference (Alt./Ref., upper) or alternative/(alternative+ reference) % (Alt./(Alt.+Ref.), lower)

To gain insight into the global patterns of psyAShM sites, we examined their distribution using ANNOVAR software, which was used to annotate the specific genomic context of the 807 psyAShM sites (Fig. 3A). We found that the majority of the identified 807 psyAShM sites were located in noncoding regions, including intronic regions (51.67%), intergenic regions (33.46%), and noncoding RNA intronic regions (4.96%). Intriguingly, 807 psyAShM sites were also significantly enriched in eQTLs and brain mQTLs, and in genes expressed specifically in the brain or blood (Fig. 3B), further supporting their potential regulatory roles. We then observed that these psyAShM sites showed a bias toward alternative allele hydroxymethylation (alt/ref=2.07 for 8544 AShM sites and 2.87 for 807 AShM sites), and this preferential pattern of alternative allele hydroxymethylation showed a significant association with psychiatric disorders. On average, alternative alleles of the 807 identified psyAShM sites were preferentially highly hydroxymethylated in affected (alt./ref.=7.3 and alt.%= 88%) compared to those in unaffected (alt./ref.=1.5 and alt.%= 60%) individuals (OR=4.8 and *P*=4.0e−30; Fig. 3C). These results suggested that psyAShM transition-induced hydroxymethylation alterations in alternative alleles might be associated with chromatin states, TF binding activity, and gene expression and ultimately affect the phenotype of SCZ/BPD.

**Fig 4:**
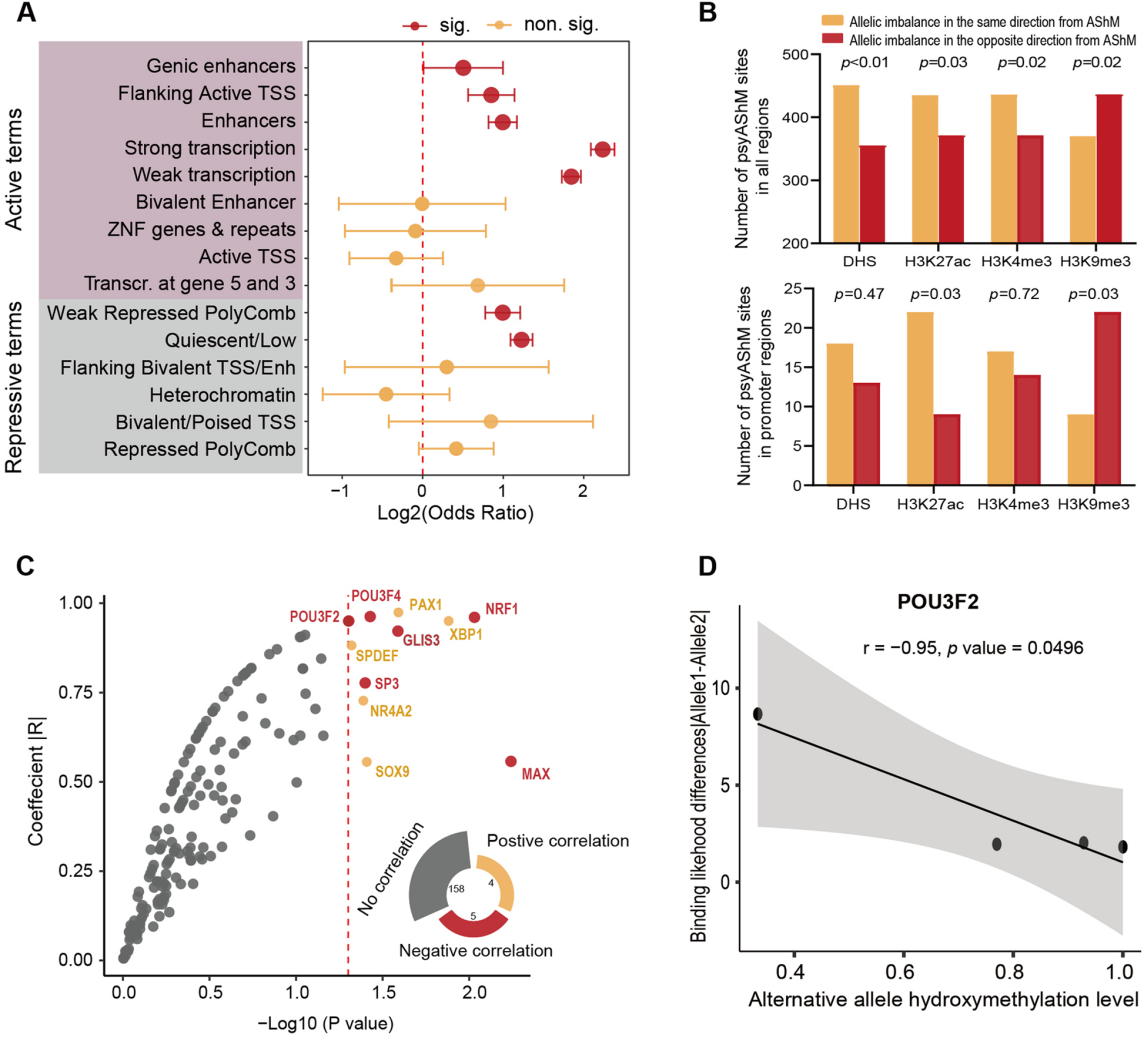
psyAShM sites exert epiallelic effects on chromatin states and TF binding affinities. **A** Enrichment of 807 psyAShM sites in chromatin states. *p*<0.05 from Fisher’s exact tests are shown with red points (log2 (OR) and 95% confidence intervals). **B** Number of psyAShM sites in the DNase I hypersensitive site (DHS) and histone marks overlapping AShM loci throughout different classes of genomic elements. The *P* value from a binomial test is shown. **C** Effects of AShM on absolute TF binding affinity based on position weight matrix scores (PWMs) at predicted TF binding sites. The negative correlation between absolute TF binding affinity differences and the alternative allele hydroxymethylation level at predicted TF binding sites is shown in red, the positive correlation between absolute TF binding affinity differences and the alterative allele hydroxymethylation level at predicted TF binding sites is shown in orange, and data showing no correlation are shown in gray. **D** Correlation between absolute POU3F2 binding affinity differences and alternative allele hydroxymethylation level at five predicted POU3F2 binding AShM sites

Next, we examined the associations of psyAShM sites with chromatin state by examining the 807 psyAShM sites that overlapped with existing ChromHMM annotations in 10 brain tissues obtained from the NIH Roadmap epigenomics consortium [24, 25]. We observed that these psyAShM sites were enriched with most activated chromatin state-associated terms (genic enhancers, flanking active TSS, enhancers, strong transcription, and weak transcription) and only with two repressive chromatin state-associated terms (weak repression polyComb and quiescent/low; Fig. 4A). Moreover, we observed that DNase I hypersensitive sites (DHS), H3K27ac and H3K4me3 marks representing active chromatin states were more abundant on the allele with more DNA hydroxymethylation of the psyAShM sites in all regions, and significantly consistent patterns were shown for H3K27ac marks enriched in promoter regions (Fig. 4B). In contrast, repressive H3K9me3 signal was more abundant on the allele with less DNA hydroxymethylation of the psyAShM sites in all regions, including promoter regions. These results indicated that the psyAShM transitions might be involved in the activation of transcription.

We further evaluated the role of epiallele-specific TF binding at 807 psyAShM sites by focusing on a set of 358 TFs that had been assessed for binding affinity using on the basis of the high-throughput systematic evolution of ligands determined by the exponential enrichment method [28]. We first identified 167 TFs that showed allele-specific binding affinity in at least three of 807 psyAShM sites and then examined the correlation between allelic differences in motif binding strength and the hydroxymethylation level of alternative alleles at these psyAShM loci for each of 167 TFs (Fig. 4C). We identified 5 TFs (PAX1, XBP1, SPDEF, NR4A2, and SOX9; orange in Fig. 4C) among the 11 TFs that showed individually significant correlations (|*r*|>0.5 and *P* <0.05; Additional file 1: Table S7 and Additional file 2: Fig. S2) with larger differences in allele-specific TF binding affinities corresponding to higher hydroxymethylation levels of alternative alleles, and 6 TFs (POU3F2, NRF1, POU3F4, GLIS3, SP3, and MAX; red in Fig. 4C) showed negative correlations, with larger differences in allele-specific TF binding affinities corresponding to lower hydroxymethylation levels of alternative alleles. For example, POU3F2 showed alternative allele-specific binding affinity across four psyASM sites, and a gain of hydroxymethylation at alternative alleles weakened its allele-specific binding affinities (Fig. 4D). Altogether, these results suggested the involvement of the psyAShM transition in gene expression alterations through its effects on epiallele-specific TF binding.

### The rs4558409 regulatory locus displays epiallelic inhibition of POU3F2 binding affinity

**Fig 5:**
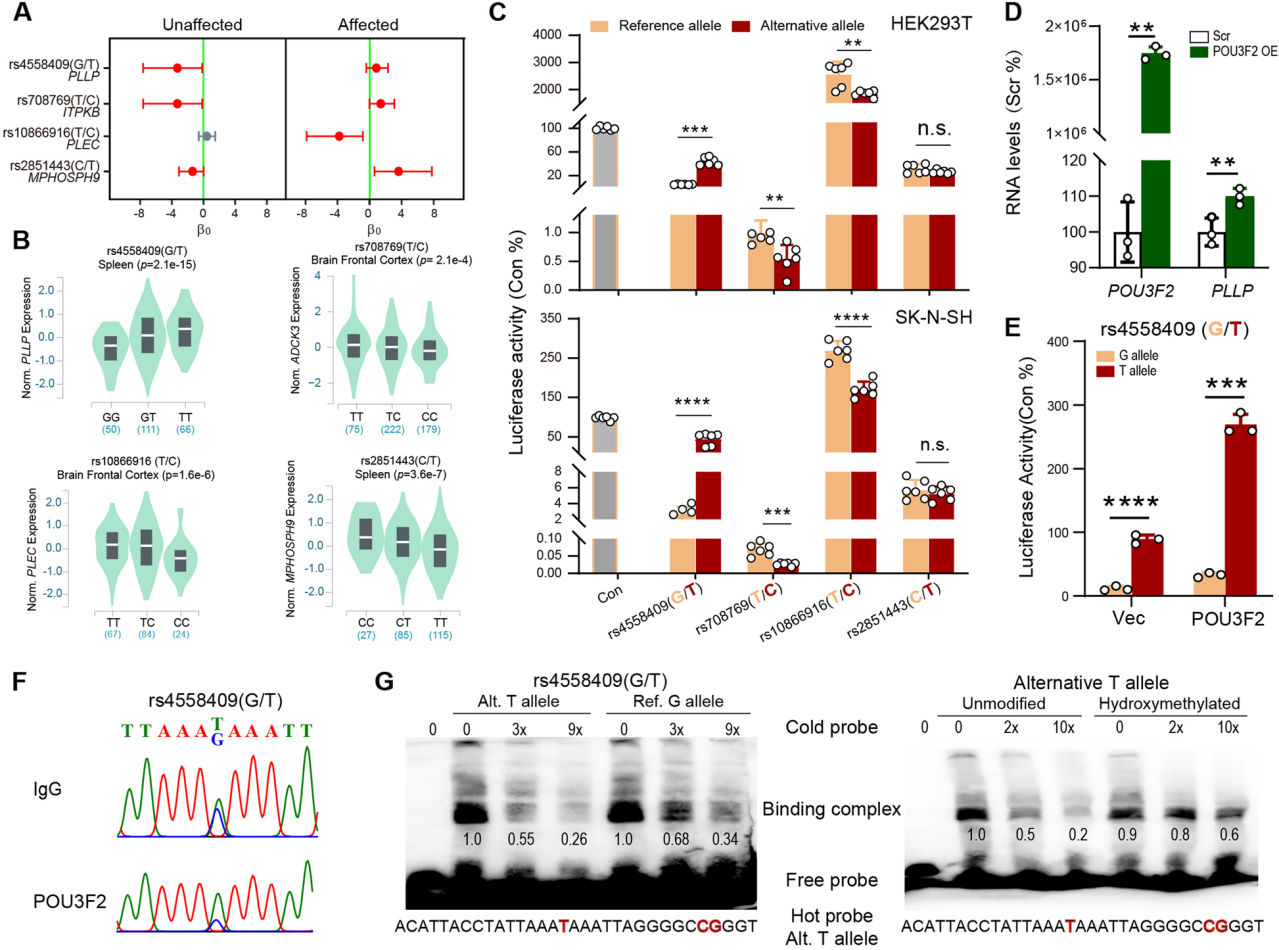
psyAShM transition exerts an allelic effect on TF binding affinity. **A** Estimates for four discordant AShM sites in PDC twin pairs. Each point represents the β0 value of the psyAShM sites showing the strongest evidence of AShM in unaffected or affected individuals (highlighted in red, *p*<1e−3) and a discordant AShM pattern in PDC twin pairs (BF>10). Error bars indicate 95% credible intervals. **B** eQTL patterns of the four psyAShM sites from the GTEx dataset. **C** Allelic effects of rs4558409 (G/T), rs708769 (T/C), rs10866916 (T/C), and rs2851443 (C/T) on promoter activity of the luciferase reporter in HEK293T and SK-N-SH cells are shown in columns with standard errors indicated by bars. The gray column represents cells transfected with pGL4.23-empty vector (Con), the orange column represents cells transfected with the reference allele-containing fragment cloned in pGL4.23, and the red column represents the alternative allele-containing fragment. **D, E** Effect of POU3F2 overexpression on endogenous *PLLP* expression (**D**) or rs4558409 allele-dependent promoter activity of the luciferase reporter (**E**) in HEK293T cells. Vec represents the pcDNA3.1 empty vector. **F** Sanger sequencing traces from ChIP-PCRs of POU3F2 and IgG at the rs4558409 (G/T) site in HEK293T cells with a heterozygous rs4558409 genotype. **G** EMSA and competition analysis. Assays were performed with the rs4558409 alternative T allele (red bold) as the hot probe, the rs4558409 alternative T allele or the reference G allele as the cold probe (left) and the alternative T allele without or with hydroxymethylated CpG (red bold) as another cold probe (right) with HEK293T nuclear extracts. Fold differences of molar excess of the cold probe compared to the hot probe and the relative intensity of the binding complex are shown underneath each panel. **p < 0.01, ***p < 0.001, ****p< 0.0001 or ns, nonsignificant from *t* test

We then sought to validate the downstream functional consequences of these psyAShM sites by predicting allelic differences in TF binding. We focused on 68 psyAShM sites embedded within the binding motifs of the 11 aforementioned TFs because each showed epiallele-specific binding affinities across these psyAShM sites (Fig. 4C). Of the 68 psyAShM sites, four sites found to be eQTLs and subthreshold SCZ or BPD GWAS SNPs, exhibiting significant AShM transitions in PDC twin pairs (Fig. 5A, B and Additional file 1: Table S8), were selected and assessed to validate their regulatory roles in SK-N-SH and HEK293T cell lines. We observed that three of the four SNPs, including *PLLP*-rs4558409 in the POU3F2 binding motif, *ITPKB*-rs708769 within the PAX1-binding motif, and *PLEC*-rs10866916 within the SP3-binding motif, showed significant allelic effects on luciferase activities in both HEK293T and SK-N-SH cell lines (Fig. 5C). These allele-specific effects were consistent with eQTL patterns observed in the GTEx or CommonMind Consortium (CMC) datasets (Fig. 5B and Additional file 1: Table S8). Since *ITPKB*-rs708769 showed very low promoter activity and PAX1 also exhibited low expression in the GTEx dataset, we then focused on the epiallele-specific effects of rs4558409 and rs10866916 on TF binding.

We observed that overexpression of *POU3F2* significantly increased the mRNA level of endogenous *PLLP* (Fig. 5D), and the alternative T allele rs4558409 exhibited greater activation of promoter activity than the reference G allele in luciferase reporters cotransfected with *POU3F2* into HEK293T cells (Fig. 5E). This result is consistent with rs4558409 showing an alternative T allele-specific POU3F2 binding affinity (Additional file 1: Table S8) and a T-allele-dependent upregulation of *PLLP* RNA, as determined based on eQTL patterns (Fig. 5B). Although we observed that SP3 showed upregulation of endogenous *PLEC* transcription and rs10866916 reference T-allele-specific promoter activity enhancement in a luciferase reporter assay after cotransfection with *SP3* in HEK293T cells (Additional file 2: Fig. S3), this finding was inconsistent with our TF binding affinity prediction results, which revealed alternative C-allele-specific SP3-binding affinity (Additional file 1: Table S8), indicating that other inhibitory TFs were involved in regulating *PLEC* expression through the rs10866916 site. We further verified that POU3F2 displayed binding activity around the rs4558409 (G/T) site (Additional file 2: Fig. S4) and showed a preference for occupying the alternative T allele over the reference G allele in POU3F2-ChIP-DNA compared with IgG-ChIP-DNA in the HEK293T cell line with heterozygous genotypes at rs4558409 (Fig. 5F). Moreover, we observed that the rs4558409 alternative T allele displayed higher binding activity than the reference G allele (Fig. 5G left), and a hydroxymethylated T allele probe displayed a lower binding activity than an unmodified probe (Fig. 5G right) and a methylated probe (Additional file 2: Fig. S5), as demonstrated via EMSA with HEK293T nuclear extracts. The AShM transition toward hyper-hydroxymethylation correlated with the rs4558409 alternative T allele under disease conditions might contribute to reduced *PLLP* expression through the inhibition of POU3F2 binding affinity. Intriguingly, rs4558409 is located in the intronic regions of *PLLP*, which has been reported to be significantly downregulated in postmortem SCZ brains (Log2FC=−0.129, *p*=0.00058), and marginally in BPD patients (Log_2_FC=−0.104, *p*=0.051), compared with that in nonpsychiatric controls in the PsychENCODE brain RNA-seq datasets [47], supporting the putative inhibitory effects of AShM transitions at rs4558409 on dysregulation of *PLLP* expression in SCZ or BPD.

### Disruption of rs4558409 promotes neural development and vesicle trafficking

**Fig 6:**
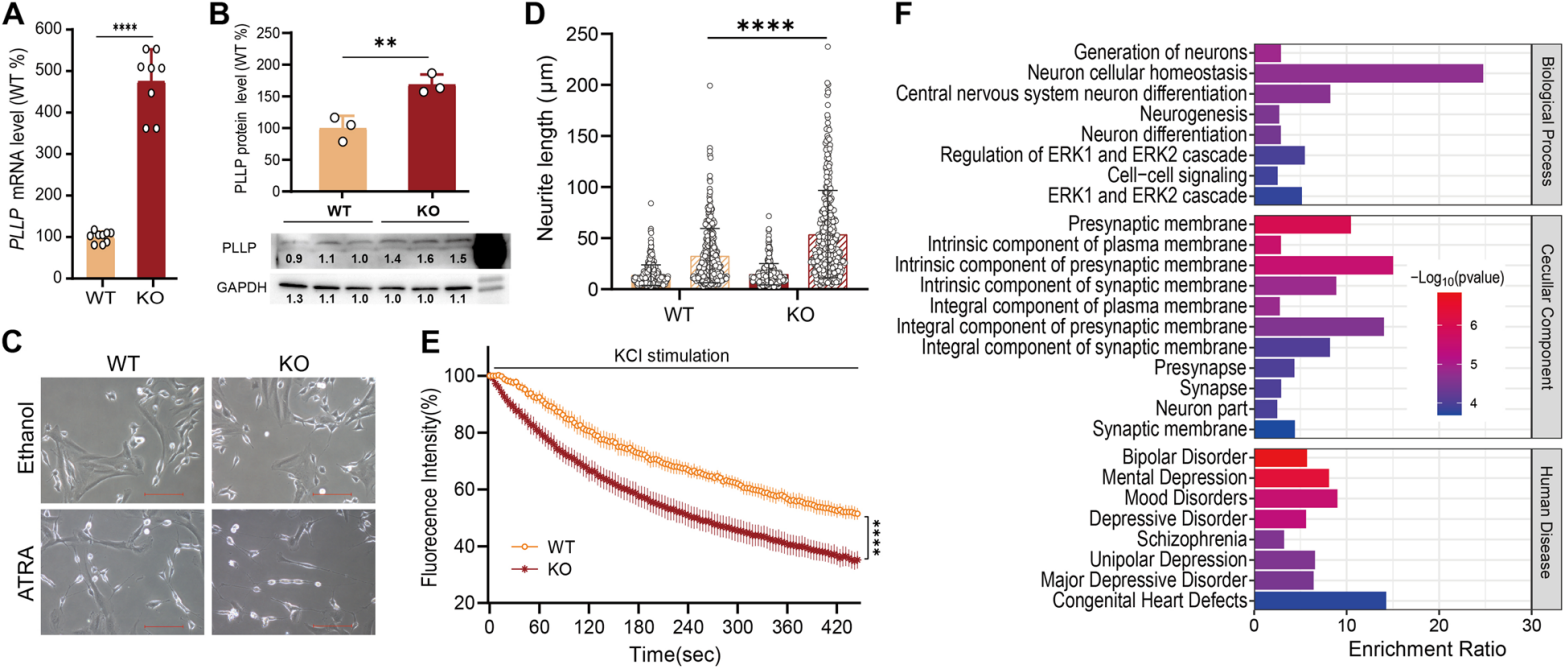
Disruption of rs4558409 promotes neural development and vesicle trafficking. **A, B** Effects of rs4558409-KO (KO) on *PLLP* RNA (**A**) and protein (**B**) expression levels in SK-N-SH cells. **p< 0.01 and ****p < 0.0001 from two-tailed Student’s *t* test. The relative intensity of binding as determined via Western blot analysis is shown underneath each band. **C** rs4558409-KO promoted neural development of SK-N-SH cells without or with ATRA treatment. The scale bar represents 100 μm. **D** Neurite-like length of the wild-type (WT, orange) or rs4558409-KO (KO, red) SK-N-SH cells without (solid) or with ATRA treatment (hatched). ****p<0.0001 from one-way ANOVA with Tukey’s multiple comparisons test. **E** FM4-64 imaging analysis of the wild-type (WT, orange) or rs4558409-KO (KO, red) SK-N-SH cells. ****p< 0.0001 obtained from two-way ANOVA. **F** Functional enrichment analysis of rs4854158-KO-induced DEGs in SK-N-SH cells

To examine the function of the rs4558409-containing region, we employed Cas9/sgRNA editing to disrupt the sequences around rs4558409 in human neuroblastoma SK-N-SH cells (rs4558409-KO; Additional file 2: Fig. S6), which has the ability to differentiate into a neuronal phenotype characterized by extensive neurite outgrowth and is the most widely used system to study neurodevelopment [3, 41–45]. We observed that rs4558409-KO significantly increased *PLLP* mRNA expression levels (increased by 475%; Fig. 6A) and protein expression levels (by 169%; Fig. 6B) in SK-SN-SH cells. *PLLP* encodes the proteolipid plasmolipin, which is a main component of synaptic plasma membranes and myelin sheaths and is involved in intracellular transport and neurite growth [49]. We then observed that rs4558409-KO promoted all-trans retinoic acid (ATRA)-induced neural development (Fig. 6C), causing an increase in the neurite-like length of SK-N-SH cells after treatment with 10 μM ATRA (Fig. 6D). Next, we monitored exocytosis using the dye FM4-64 in the presence of 75 mM KCl to examine whether rs4558409-KO has a direct effect on vesicle secretion from KCl-induced SK-N-SH cells (Additional file 2: Fig. S7A). We observed that FM4-64 was depleted faster in rs4558409-KO cells than in unperturbed SK-N-SH cells (Fig. 6E and Additional file 2: Fig. S7B), indicating enhanced exocytosis driven by rs4558409-KO.

We also examined the transcriptome profiles of rs4854158-KO SK-N-SH cells without ATRA treatment by RNA-seq and identified 1115 DEGs (|Log_2_FC|>1 and FDR<0.1) including 56 upregulated DEGs and 59 downregulated DEGs in rs4854158-KO cells (Additional file 1: Table S9). Functional enrichment analysis revealed that GO-BP such as generation of neurons, central nervous system neuron differentiation, neurogenesis, and neuron differentiation; GO-Cellular Component such as presynaptic membrane, synapse, neuron part, and synaptic membrane; and human diseases such as BPD, SCZ, and MDD were significantly enriched among rs4854158-KO-induced DEGs (Fig. 6F and Additional file 1: Table S10). Although *PLLP* did not show a significant increase (Log2FC = 0.2289, *p* = 0.6264) in rs4854158-KO cells, the upregulated *ERBB4* (Erb-B2 Receptor Tyrosine Kinase 4) and *NRG1* (Neuregulin 1) and the downregulated *GAD1* (Glutamate Decarboxylase 1) in rs4854158-KO cells, which are involved in neural development, may partially explain the altered neural development in rs4854158-KO cells. These genes have also been reported to associated with SCZ or BPD [50, 51].

## Discussion

Here, we performed a genome-wide examination of AShM sites from MZ twins and identified a large number of SNPs showing AShM transitions across MZ twins with discordant phenotypes. These psyAShM sites displayed epiallele-specific effects on chromatin states and TF binding, and their AShM transitions may have led to dysregulated gene expression and functions and may have eventually increased the risk of SCZ or BPD. We then employed multiple lines of data to show that competitive binding of POU3F2 on the alternative T allele at the psyAShM site rs4558409 (G/T) can enhance *PLLP* expression, while the hydroxymethylated alternative allele alleviating the POU3F2 binding activity at the rs4558409 site might be associated with the downregulated *PLLP* expression observed in patients with BPD/SCZ. This study has etiological implications for the AShM transition in patients with SCZ and BPD.

Our findings showed that sensitivity of the hydroxymethylome to genetic variation among MZ twin pairs with discordant phenotypes displayed disease-associated features through epiallele-specific effects on chromatin states, TF binding, and gene expression. Our allelic epigenome profiling of 17 MZ twin pairs revealed that 1.2% of informative SNPs showed AShM (FDR<0.1), which was a more conservative outcome than estimates for ASM (2.2%) in the same twin pairs analyses in our previous study [3]. Some of our identified AShM sites (11.7%) displayed sequence-dependent ASM, as shown in our previous study [3], and were located in previously reported imprinted regions. The regulatory role of AShM sites was shown by the evidence of their uneven distribution across the genome with 90% of the psyAShM sites located in noncoding regions, greater enrichment of pysAShM sites in the active chromatin state, and AShM transitions that may induce changes in chromatin state and TF binding ability, eventually inducing altered gene expression. The regulatory roles of psyAShM were further demonstrated by their significant enrichment in brain eQTLs/mQTLs, SCZ/BPD-associated DEGs, and TWAS or GWAS of SCZ/BPD. In fact, we recently reported that ASM sites are more enriched in repressed chromatin states and showed epiallelic effects on TF binding and gene expression [3]. DNA methylation has long been associated with gene silencing, inferring that DNA demethylation can lead to gene activation. As an intermediate of DNA demethylation processing, 5hmC has also been reported to be highly enriched in the gene bodies of transcriptionally active genes, promoters, and enhancers and to undergo highly dynamic changes during development and differentiation and in the context of neuropsychiatric disorders [10, 12]. The functional implication of sequence-dependent ASM and AShM sites examined in our study, together with mostly functional genetic variants identified in noncoding regions via GWAS [2], provided further evidence for dynamic DNA methylation/demethylation in mediating associations among genetic risk burden, environmental or epigenetic risk exposure, and phenotype.

Our study validated the biological role of AShM transitions at the *PLLP* rs4558409 locus, leading to alterations in POU3F2 binding ability and contributing to the downregulation of *PLLP*, providing etiological implications for SCZ or BPD. *PLLP* encodes the proteolipid plasmolipin, which is a main component of synaptic plasma membranes and myelin sheaths and is involved in intracellular transport and neurite growth [49]. AShM transitions at the *PLLP* rs4558409 locus across PDC twin pairs reduced *PLLP* transcriptional activity through hydroxymethylation-mediated inhibition of active POU3F2 binding affinity at the active T allele of the rs4558409 (G/T) regulatory locus. The allele-specific regulatory role of rs4558409 in a luciferase reporter assay was consistent with the eQTL pattern. The alternative T allele of rs4558409 showing higher binding activity was demonstrated via ChIP and EMSAs, and its hyper-hydroxymethylation/methylation, which reduced binding ability, might significantly contribute to the reduced *PLLP* expression observed in postmortem SCZ brain[47], and rs4558409 disruption promotes neural development and vesicle trafficking. Similarly, reduced *PLLP* has been observed in the temporal cortex of patients with MDD [52]. In fact, rs4558409 is a subthreshold GWAS SNP associated with SCZ (*p*=0.007479 in PGC3) [36] and MDD (*p*=0.026) [40], and hyper-hydroxymethylation at the risk alternative T allele of rs4558409 (G/T), which reduced TF binding affinity and *PLLP* expression, might increase disease susceptibility. PLLP plays an important role in membrane biogenesis and myelination [49], and distorted oligodendrocyte differentiation and subsequent defects in myelination might lead to SCZ and BPD onset [49, 53]. Intriguingly, *POU3F2* is one of the key regulators coexpressed with many genes within the SCZ-related module [54], and *POU3F2*-regulated target genes may contribute to neurodevelopment and synaptic function in various ways [55]. In addition, POU3F2-deficient mice exhibited impaired hippocampal neurogenesis [56]. *POU3F2* expression is decreased in cerebral organoids in patients with BPD [57], and its expression is associated with differential SCZ-associated alterations in brain tissues, including downregulation in the SZDB dataset [58] but upregulation in the PsychENCODE dataset [47]. In summary, involvement of AShM transitions at rs4558409 regulatory loci might contribute to disease-associated *PLLP* downregulation through epiallele-specific inhibition of POU3F2 binding.

This study also has several limitations. First, although our study identified that disruption of rs4558409-contained region promotes neural development and vesicle trafficking, whether rs4558409 (G/T) display allelic effects on neural development and vesicle trafficking remains to be further examined through single-base mutation strategy in SK-N-SH cells. Second, whether KO of *PLLP* could restore neural development induced by rs4558409-KO may be needed to validate the role of rs4558409 in regulation of PLLP. Third, whether *ERBB4*, *NRG1*, or *GAD1* except for *PLLP* are regulated by rs4854158 may provide further mechanism of rs4854158 on regulation of neural development since expression of those genes are significantly dysregulated in rs4558409-KO cells. Finally, despite the human neuroblastoma SK-N-SH cell lines employed in this study are the most widely applied and cited in vitro system for neurodevelopmental and neuropsychiatric studies, further studies in other appropriate test models to validate the function of rs4854158 in pathogenesis of the BPD or SCZ are needed.

## Conclusions

We observed that the allelic imbalances in hydroxymethylation vary across MZ twin pairs with discordant phenotypes were associated with SCZ/BPD-associated features by altering gene regulation, contributing to our understanding of the interaction between genetic and epigenetic factors in mediating individual differences in disease susceptibility and providing a powerful strategy for prioritizing SCZ/BPD-associated genes or variants.

### Supplementary Information

**Additional file 1: Table S1. **Summary of 5hmC-seq and WGS data for each twin pair. **Table S2. **Primers and probes employed in this study. **Table S3. **The number of AShM sites identified in MZ twins. **Table S4. **Detailed information about 534 AShM sites in 53 previous reported known imprinted clusters. **Table S5. **Information about 807 AShM sites. **Table S6. **Functional enrichment of GO biological process (BP) annotations among 807 AShM sites annotated genes. **Table S7. **Information of allele-specific transcript factor (TF) binding affinity of 807 psyAShM sites.** Table S8. **Detailed information of four AShM sites. **Table S9. **rs4558409-KO induced DEGs in SK-N-SH cells. **Table S10. **GO-BP, GO-CC and human disease enrichment results of rs4558409-KO induced DEGs.

**Additional file 2: Fig. S1. **Genome-wide DNA hydroxymethylation patterns of MZ twins.** Fig. S2. **Correlation analysis results.** Fig. S3. **Allelic effects of rs10866916 on the SP3 activity. **Fig. S4. **ChIP-qPCR.** Fig. S5. **EMSA and competition analysis.** Fig. S6. **Sequencing chromatographs of PCR products of rs4558409-dirupted PLLP in SK-N-SH cells. **Fig. S7. **FM4-64 imaging analysis of neurite length.

## Data Availability

All the data generated in this study were shown in the main text and additional files. RNA sequencing data of rs4854158-KO SK-NSH cells have been deposited to the NCBI GEO site with the GEO number GSE246593. Additional information is also available upon reasonable request to the corresponding authors.

## Abbreviations

5hmC: 5-Hydroxymethylcytosine
AD: Alzheimer disease
AShM: Allele-specific hydroxymethylation
ASM: Allele-specific methylation
ASoC: Allele-specific open chromatin
ATAC-seq: Assay for transposase-accessible chromatin using sequencing
ATRA: All-trans retinoic acid
BCC: BPD-concordant twins
BDC: BPD-discordant twins
BF: Bayes factors
BPD: Bipolar disorder
ChIP: Chromatin immunoprecipitation
CMC: CommonMind Consortium
DEG: Differentially expressed genes
EMSA: Electrophoretic mobility shift assay
eQTL: Expression quantitative trait loci
ESCs: Embryonic stem cells
FC: Fold-changes
FMRP: Fragile X mental retardation protein
GTEx: Genotype-Tissue Expression
GWAS: Genome-wide association study
HCC: Healthy concordant twins
KO: Knock out
LCLs: Lymphoblastoid cell lines
LIBD: Lieber institute for brain development
MDD: Major depressive disorder
MZ: Monozygotic
OR: Odd ratio
PCC: Psychiatric disorder-concordant twins
PDC: Psychiatric disorders in discordant MZ twins
*PLLP*: Plasmolipin
*POU3F2*: POU Class 3 Homeobox 2
PSD: Postsynaptic density
psyAShM sites: Psychiatric disorder-associated AShM sites
SCC: SCZ-concordant twins
SCZ: Schizophrenia
SDC: SCZ-discordant twins
SNPs: Single-nucleotide polymorphisms
TET: Ten eleven translocation
TFs: Transcription factors
TSS: Transcription start sites
TWAS: Transcriptome-wide association study
WGS: Whole-genome sequencing
