# Supplementary material for: Transition of allele-specific DNA hydroxymethylation at regulatory loci is associated with phenotypic variation in monozygotic twins discordant for psychiatric disorders
Source: BMC Med. 2023 Dec 12;21:491. doi: 10.1186/s12916-023-03177-y (PMC10714646; doi:10.1186/s12916-023-03177-y)
Supplement: Supplementary file 2 — Additional file 2: Fig. S1. Genome-wide DNA hydroxymethylation patterns of MZ twins. Fig. S2. Correlation analysis results. Fig. S3. Allelic effects of rs10866916 on the SP3 activity. Fig. S4. ChIP-qPCR. Fig. S5. EMSA and competition analysis. Fig. S6. Sequencing chromatographs of PCR products of rs4558409-dirupted PLLP in SK-N-SH cells. Fig. S7. FM4-64 imaging analysis of neurite length. [file 12916_2023_3177_MOESM2_ESM.docx]

**Additional file 2**

**Supplementary figures**


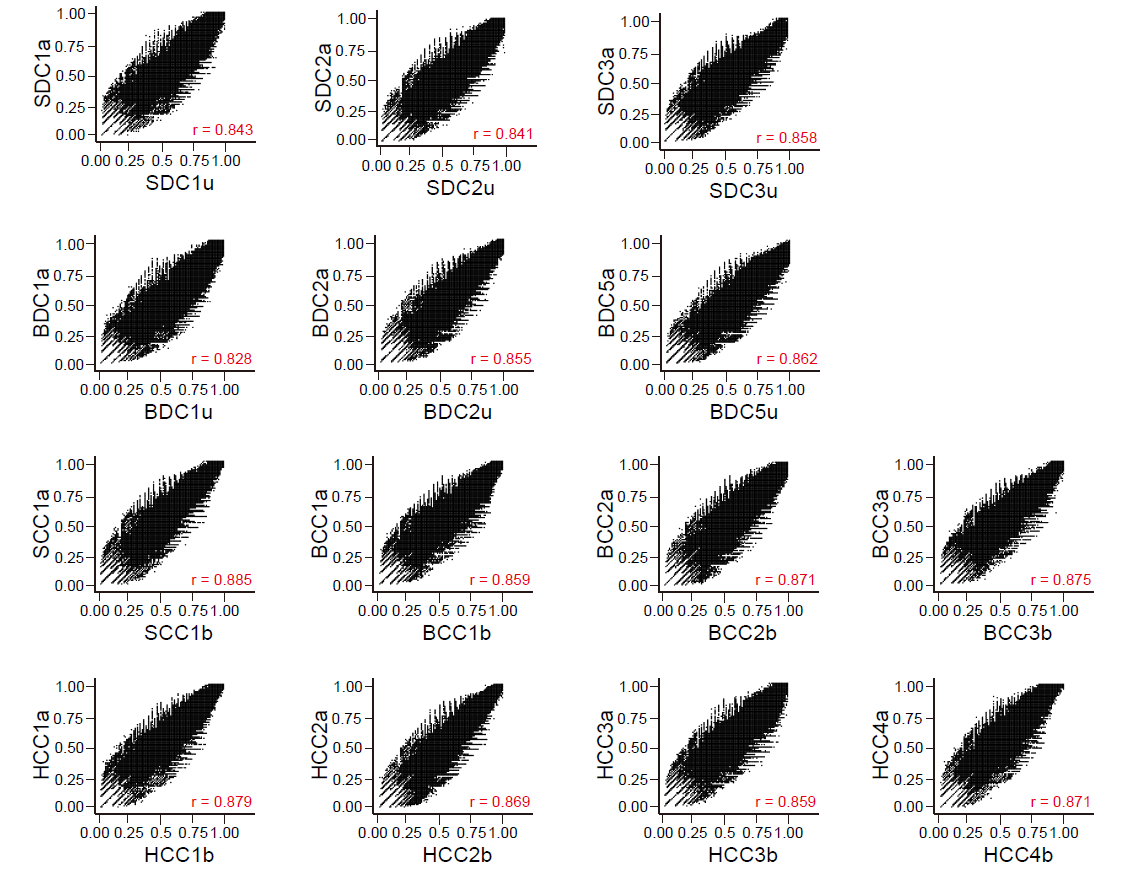


**Figure S1 Genome-wide DNA hydroxymethylation patterns of MZ twins.** Each dot in the plots represents the relative methylation score (RMS) of each 500-bp bin from each of two individuals within one twin pair. The r value represents the Pearson's r correlation within-twin.

**
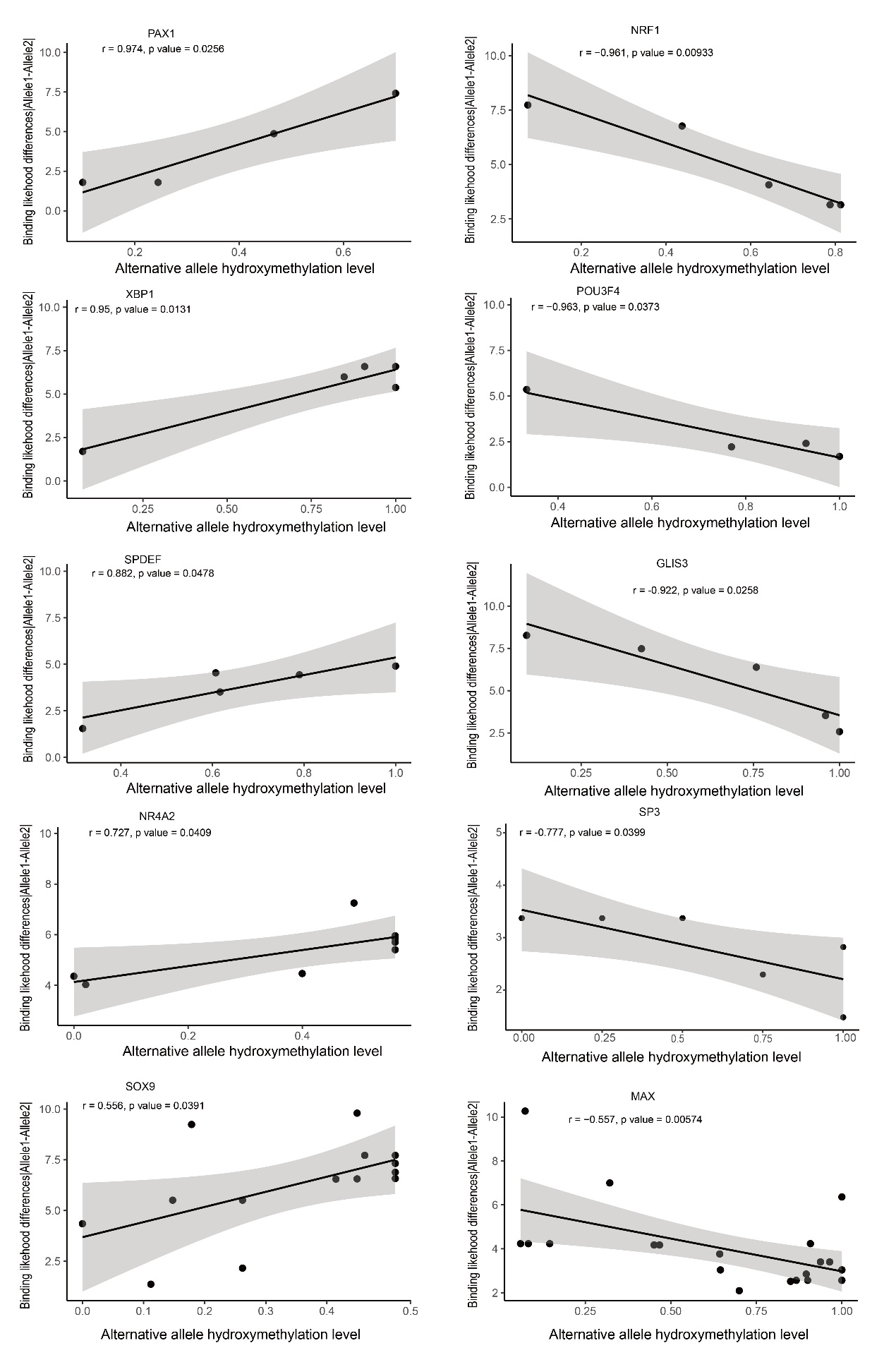
**

**Figure S2 Correlation analysis results.** Correlation between absolute TF binding affinity differences and the alternative allele hydroxymethylation level of AShM sites at predicated TF binding sites.

**
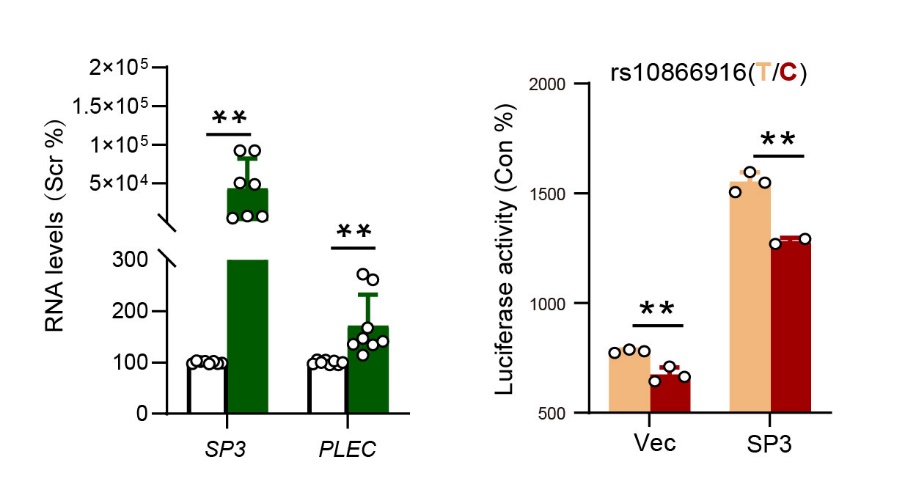
**

**Figure S3** **Allelic effects of rs10866916 on the SP3 activity.** (**Left**) Effect of SP3 overexpression on the *PLEC* RNA expression level in HEK293T cells. White column represents cells transfected with pcDNA3.1 empty vector, and green column represents cells transfected with SP3 cloned in pcDNA3.1 vector. (**Right**) Allelic effects of rs10866916 on the promoter activities of luciferase reporter in HEK293T cells overexpressed SP3 in pcDNA3.1 vector. The orange column represents cells transfected with the reference allele-containing fragment cloned in pGL4.23 and the red column represents the alternative allele-containing fragment. Vec represents the pcDNA3.1 empty vector. ***P* < 0.01 from *t* test.

**
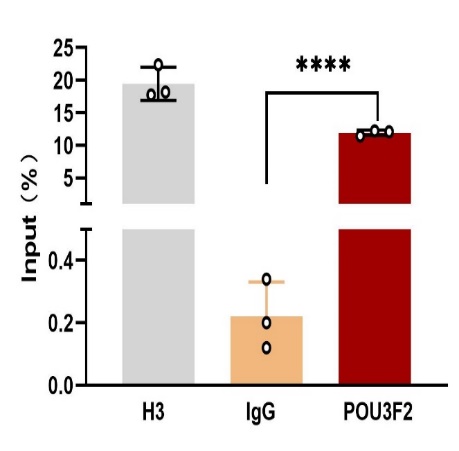
**

**Figure S4 ChIP-qPCR.** ChIP-qPCR measurement of POU3F2, and H3 histone as positive control and IgG as negative control, occupancy of rs4558409 (G/T) regulatory locus in HEK293T cells. Data indicate mean ± SD. *****P* < 0.0001 from *t* test

**
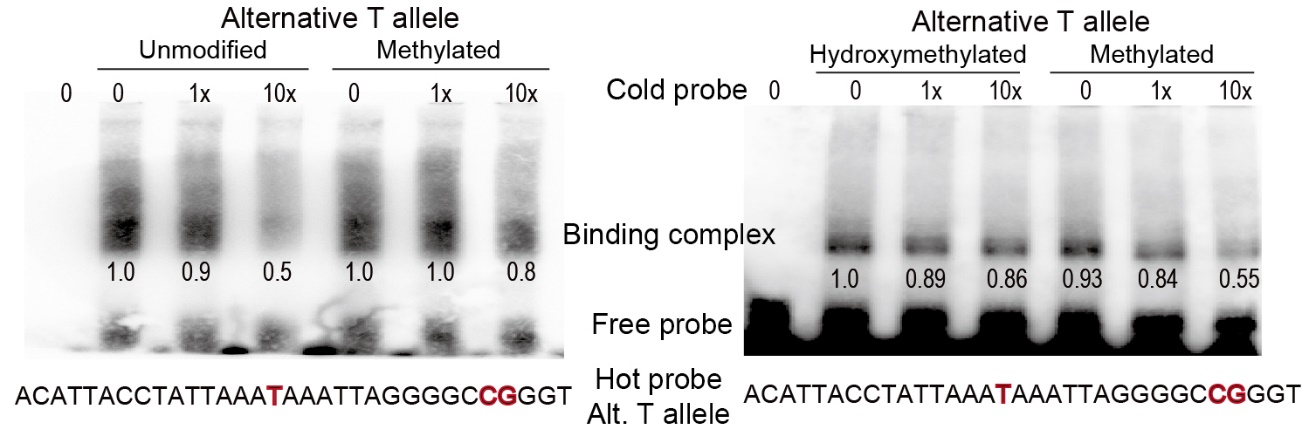
**

**Figure S5 EMSA and competition analysis.** Assays were performed with the rs4558409 alternative T allele as the hot probe, the rs4558409 alternative T allele without modified CpG or with methylated CpG as the cold probe (left) and the alternative T allele with hydroxymethylated CpG or with methylated CpG as another cold probe (right) with HEK293T nuclear extracts. Fold differences of molar excess of the cold probe compared to the hot probe and the relative intensity of the binding complex are shown underneath each panel.

**
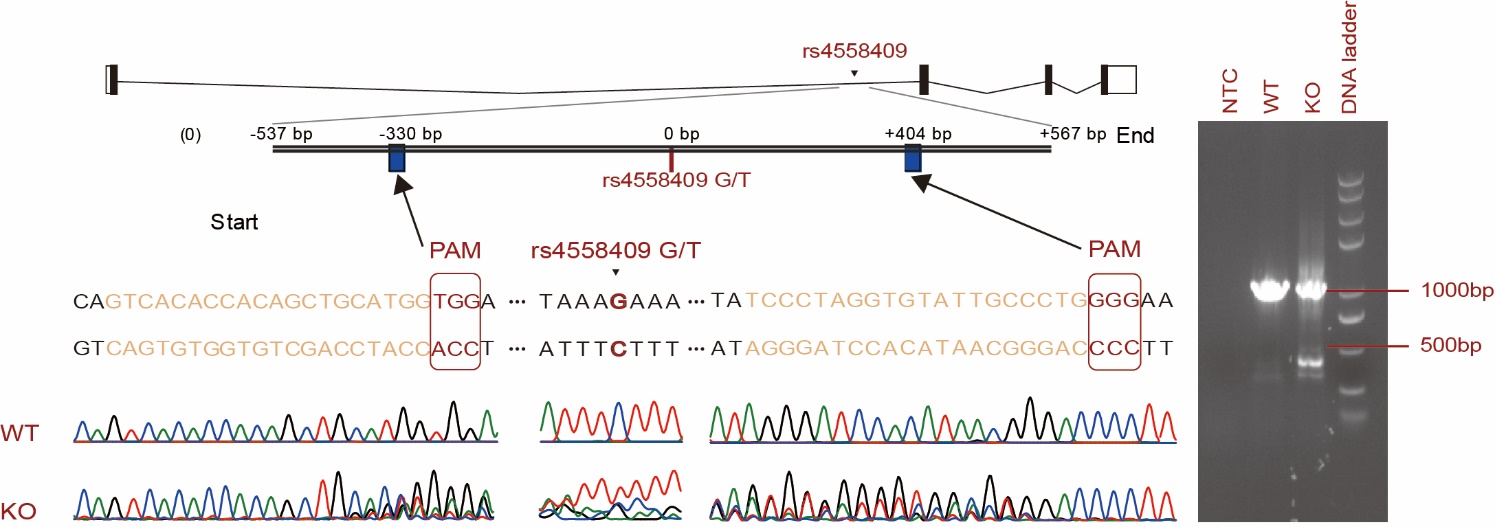
**

**Figure S6** Sequencing chromatographs of PCR products of rs4558409-dirupted *PLLP* in SK-N-SH cells.

**
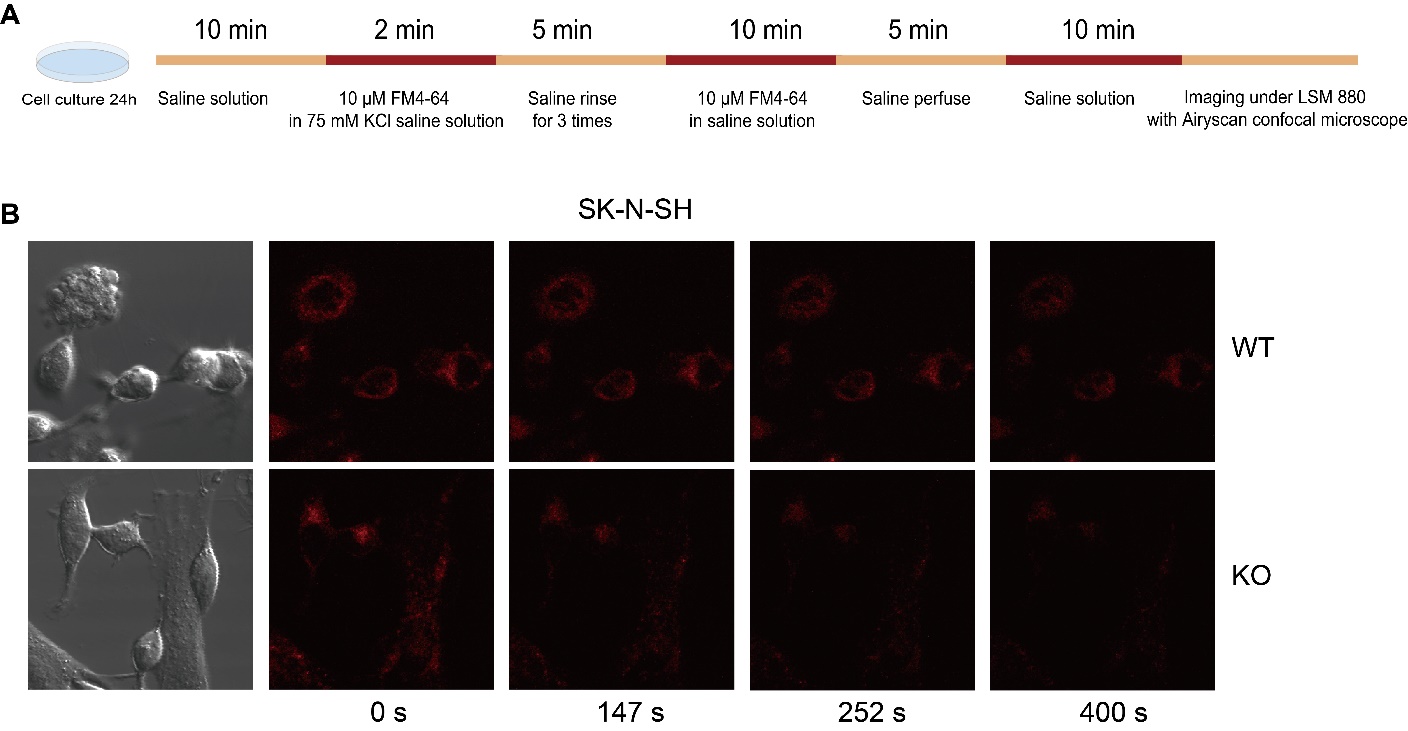
**

**Figure S7** **FM4-64 imaging analysis of neurite length. (A)** Illustration of procedure for cell treatment before FM4-64 imaging. **(B)** Representative images from FM4-64 imaging analysis of the wild-type (WT) or rs4558409-KO (KO) SK-N-SH cells at 0, 147, 252 and 400 second (s) after KCI stimulation.
